# A Stochastic Model for CD4+ T Cell Proliferation and Dissemination Network in Primary Immune Response

**DOI:** 10.1371/journal.pone.0135787

**Published:** 2015-08-24

**Authors:** Alessandro Boianelli, Elena Pettini, Gennaro Prota, Donata Medaglini, Antonio Vicino

**Affiliations:** 1 Systems Medicine of Infectious Diseases group and Braunschweig Integrated Centre of Systems Biology, Department of Systems Immunology, Helmholtz Centre for Infection Research, Inhoffenstrasse 7, 38124 Braunschweig, Germany; 2 Laboratorio di Microbiologia Molecolare e Biotecnologie, Dipartimento di Biotecnologie Mediche, Università di Siena, Viale Bracci 1, 53100 Siena, Italy; 3 Dipartimento di Ingegneria dell’Informazione e Science Matematiche, Università di Siena, Via Roma 56, 53100 Siena, Italy; Jackson Laboratory, UNITED STATES

## Abstract

The study of the initial phase of the adaptive immune response after first antigen encounter provides essential information on the magnitude and quality of the immune response. This phase is characterized by proliferation and dissemination of T cells in the lymphoid organs. Modeling and identifying the key features of this phenomenon may provide a useful tool for the analysis and prediction of the effects of immunization. This knowledge can be effectively exploited in vaccinology, where it is of interest to evaluate and compare the responses to different vaccine formulations. The objective of this paper is to construct a stochastic model based on branching process theory, for the dissemination network of antigen-specific CD4+ T cells. The devised model is validated on *in vivo* animal experimental data. The model presented has been applied to the vaccine immunization context making references to simple proliferation laws that take into account division, death and quiescence, but it can also be applied to any context where it is of interest to study the dynamic evolution of a population.

## Introduction

The analysis of regulating mechanisms underlying T cell activation, division, death, differentiation and dissemination represents a fundamental issue in numerous contexts of cell biology. The initial phase of the adaptive immune response after first antigen encounter, known as immune priming, is a critical event that strongly affects the magnitude and quality of the immune response. It is markedly characterized by T cell proliferation and dissemination processes ([1],[2]). Priming of T helper cells represents a key step in vaccination due to the close relationship between CD4+ T cells and long term immunity [3]. Generation of primed T cells requires contact between antigen presenting cells and specific T helper cells within lymph nodes, thus leading to T cell proliferation. CD4+ T cells undergo numerous rounds of division expanding the population of antigen specific T helper cells that are able to interact with and regulate B cell immune function [4]. T cell primary activation indeed influences both B and T cell memory generation, thus determining the success of a vaccination strategy. In fact, recent studies have shown that the frequency of Ag-specific primed CD4+ T cells can predict the intensity of the secondary humoral responses ([5],[6]).

Characterization of the T-cell priming properties of different vaccine formulations is essential for the rational design of effective prime-boost combinations [7]. The heterologous prime-boost approach is aimed at the generation and enrichment of antigen-specific B and T-cell responses required to fight a specific pathogen ([8],[9]).

Since only a few of the essential features characterizing proliferation and dissemination of T cells can be directly obtained by experimental measurements, mathematical models represent an attractive tool for estimating biologically meaningful parameters determining proliferation, trafficking and death of the T cell population after vaccination ([10, 11]). A further added value of a validated model is to provide a simulation tool capable of predicting the priming properties of different vaccination strategies. Moreover these models could be also adapted to human studies to rapidly predict vaccine immunogenicity and distinguish between responders and non responders. Quantifying the dynamics of T cells in terms of division, dissemination and death is indeed extremely important for the evaluation and comparison of vaccine candidates, adjuvants, vectors, immunization routes and for testing biological hypotheses regarding the T cell population. In recent years, experimental studies on T cell proliferation both *in vivo* and *in vitro* have benefited from the development of methods to measure the number of divisions that a T cell undergoes. One of the most informative techniques for characterizing the kinetics of T cells in the immune system is the vital dye 5-(and 6-) carboxyfluoroscein diacetate succinimidyl ester (CFSE) labeling ([12],[13]), that stains T cells very stably and it can be used to monitor lymphocyte proliferation due to the progressive halving of the dye fluorescence following cell division. Mathematical models have been used widely and successfully for the analysis of *in vitro* CFSE proliferation data ([14],[15],[16],[17],[18],[19],[20],[21],[22],[23]). For instance, in [14] a multi-type Galton Watson (MGW) process is applied for the T cell proliferation and a quasi likelihood approach is used on CFSE cell count data in order to obtain proliferation rate estimates. Although the stochastic and deterministic models used in these works capture realistic aspects of the T cell proliferation process, their direct application to *in vivo* studies would require neglecting several important phenomena, like for example the spatial dissemination of cells in the lymphoid organs, which would prevent the model from capturing the huge increase of complexity implicit in the real experimental setup. On the other hand, these intriguing aspects, which are discussed in the following paragraphs, make it rather difficult to adopt sophisticated stochastic models, like that proposed in [18], which take into account the competitive inhibition of T cell proliferation and possible differentiation.

The aim of this contribution is to address the main problems encountered when dealing with *in vivo* experiments, having no counterpart in *in vitro* studies and to propose possible modelling techniques to solve them.

It is known that the draining lymph node is where primed T cells can firstly be detected after immunization and it is also known that in this organ the division process starts. However, we know that the draining lymph node is just one of the compartments of the complex network composed of draining and distal lymph nodes, blood, lymphatic vessels and lymphoid organs. Actually, T cell proliferation starts in the draining lymph nodes, from where T cells migrate to lymphoid organs. Moreover, in the first time steps after immunization, *naive* T cells are recruited from the entire network to the draining lymph nodes. Both in- and out- migration rates, which cannot be measured directly, increase the complexity of the dynamics of the T cell population. This situation makes the construction of a comprehensive and physiologically meaningful mathematical model an extremely challenging problem. The picture becomes even more challenging if one takes into account that an important feature which characterize T cells in the decision-making process leading to further division, migration or apoptosis, is the number of divisions it has undergone, i.e., the generation number after the initial stimulus. Actually, fate determination is strictly related to the generation number of the lymphocyte (see [24], [14] and references therein). This calls for model structures providing the flexibility of considering different cell generation or cells with time varying proliferation parameters. Only few studies have been applied to *in vivo* experiments, and these involve only draining lymph nodes ([14],[25],[26],[27]). We have indeed recently applied a MGW to model *in vivo* T-cell proliferation in draining lymph nodes following vaginal immunisation ([28],[29]), however very little is known on how to model the entire dissemination network, taking into account the cell aging features. For example, the interesting paper [30] proposes a mechanistic third order state equation model of the CD8+ T cell population evolution (irrespective of T cell generations) and trafficking during influenza infection, involving lung, spleen and mediastinal lymph node. The model identified on real data from mice allowed the authors to assess that spleen represents the main source of effector T cells in the lung. Further complexity is added to the picture, if one considers the fact that the fate of primed lymphocytes is also subject to competitive inhibition regulated by a broad range of conditions such as cell-cell interactions, secreted cytokines and external signals in general (see e.g. [31] and references therein). An additional challenging element of complexity of an *in vivo* setup is the necessity of sacrificing animals to collect data. This means that measurements taken at different time points do not refer to the same individual, rather they refer to different individuals. This introduces an inter-individual heterogeneity aspect which must be properly taken into account when estimating and validating the mathematical model.

The objective of the present work is to propose a model capable of reproducing both the dynamic evolution and the spatial dissemination of the *in vivo* T cell population in the various compartments upon vaccination. A stochastic model based on the theory of branching processes has been constructed, by including in its representation immigration and migration processes which characterize the cell population dissemination. This allowed us to model the cell dissemination network in an effective way, at the same time keeping both the model computational complexity and its comprehensibility at acceptable levels. We have chosen a stochastic setting mainly because the results from several experiments [11], show that the intrinsic variances of the cell counts at different time points can be hardly explained through the introduction of an artificial external noise. Moreover, a stochastic approach naturally lends itself to possible extensions of the model adopted here, to take into account cell-cell interaction and differentiation.

## Materials and Methods

### Mice

Nine week old female OT-II TCR-transgenic mice (H-2b) and C57BL/6J mice were purchased from Charles River (Lecco, Italy). Animals were maintained under specific pathogen-free conditions in the animal facilities at the University of Siena, and treated according to national guidelines (Decreto Legislativo January 27, 1992 n. 116, implementing 86/609/CEE Directive). All animal studies were approved by the Ethics Committee “Comitato Etico Locale dell’Azienda Ospedaliera Universitaria Senese” and the Italian Ministry of Health (number 4/2011, July 20, 2011).

### Adoptive transfer of transgenic CD4+ T cells

Single cell suspensions from the spleen and pooled lymph nodes (cervical, brachial, axillary, mesenteric and iliac lymph nodes) of OT-II transgenic mice were enriched for CD4 + T cells, by negative selection using the Easy-Sep magnetic nanoparticles (StemCell Technologies, Vancouver, BC, Canada), according to the manufacturer’s protocol. The purity of the CD4+ T cell population in the enriched fraction was > 95%, as determined by flow cytometric analysis. CD4 + isolated T cells were pooled and stained with carboxy-fluorescein diacetate succinimidyl ester (CFSE, 7.5 *μM*, Invitrogen) [32], for 10 min at 37 ℃. An amount of 2.5 10^6^ of CFSE-labelled CD4+ T cells was injected into the tail vein of each recipient mouse.

### Nasal immunization of mice and sample collection

Twenty-four hours after adoptive transfer of CFSE-labelled OT-II CD4+ T cells, C57BL/6J mice were nasally immunized with OVA grade V (Sigma-Aldrich) (25 *μ*g/mouse) and CpG ODN 1826 (20 *μ*g/mouse). Mice were lightly anaesthetized by intraperitoneal injection of tiletamine and zolazepam hydrochloride (Zoletil 20, Laboratoires Virbac, France, 6 mg/kg) and xylazine (Xilor 2 per cent, Bio 98 Srl, Italy, 3 mg/kg) and then inoculated with OVA and CpG into the nostrils with a volume of 15 *μ*l. Groups of five mice were sacrificed 0, 57, 72, 84 and 96 hours following immunization. Lymph nodes draining the nasal immunisation route, iliac, and mesenteric lymph nodes and spleen were individually harvested from each mouse. Single-cell suspensions from lymph nodes and spleens were obtained as previously described [33].

### Flow cytometric analysis

Cell suspensions from lymph nodes and spleens were incubated with Fc-blocking solution [0.5 mg *CD*16/*CD*32 mAb (clone 93) (eBioscience, USA), 5% mouse serum, 5% rat serum, 0.2% sodium azide (all from Sigma-Aldrich) in 100 ml of HBSS] for 30 min at 4 ℃. Cells were stained with PerCP-conjugated anti-mouse CD4 (clone RM 4-5) (BD Pharmingen) for 30 min at 4 ℃. Count Bright absolute counting beads (Invitrogen, Molecular Probes, Oregon, USA) were used for counting the number of lymphocytes in each sample. All samples were analyzed by flow cytometry (FACScalibur, Becton Dickinson, San Diego, CA) on individual samples collected from each animal. Data analysis was performed by using Flow Jo software (Tree Star, Ashland, OR, USA).

## Results

### CD4+ T cell proliferation experimental data

**Fig 1:**
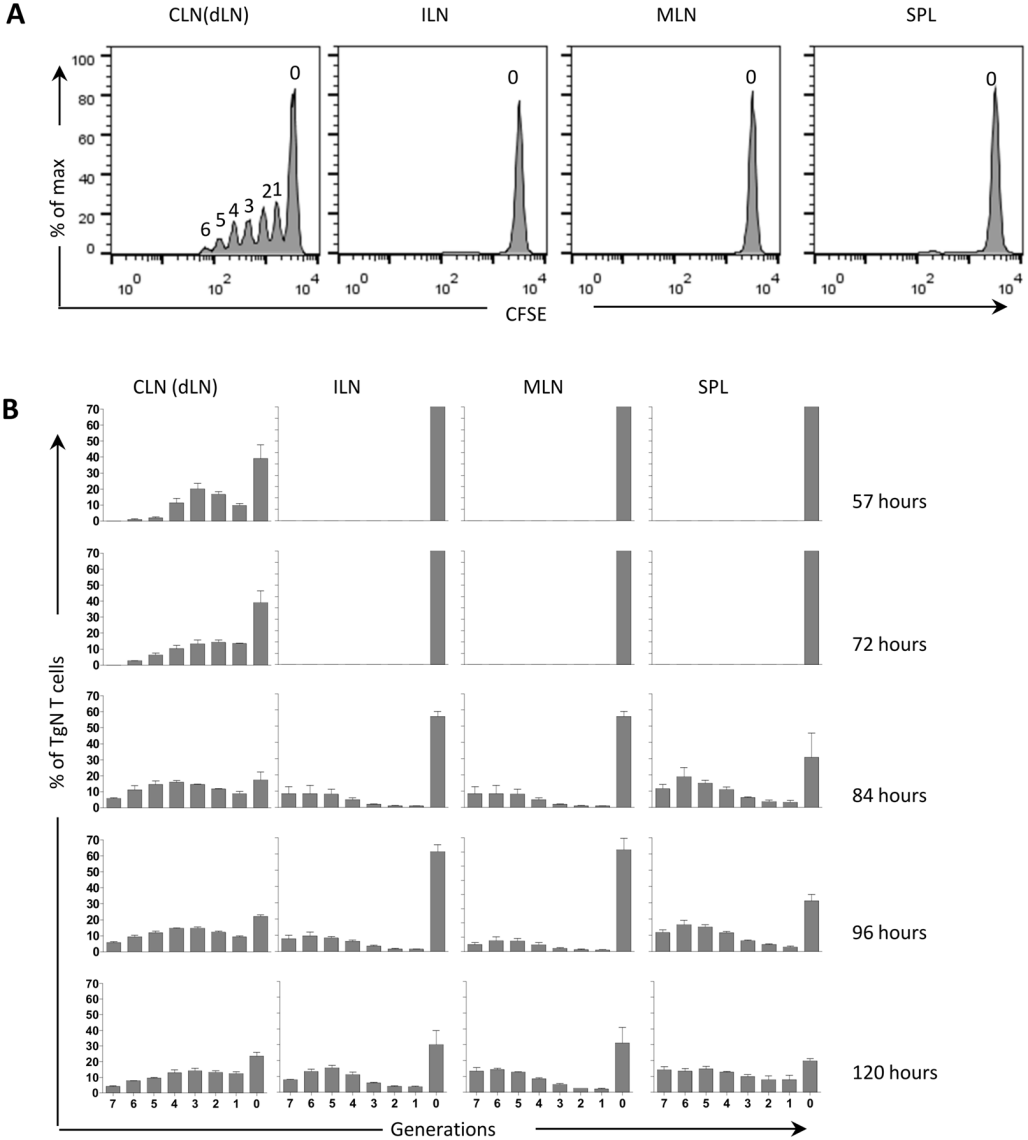
Dissemination of primed antigen-specific T cells after nasal immunization with OVA and CpG ODN 1826. CD4+ T cells, isolated from OT-II mice, were labeled with CFSE and adoptively transferred into recipient C57BL/6J mice. Twenty-four hours later, recipient mice were immunized with OVA (25 *μ*g/mouse) and CpG ODN (20 *μ*g/mouse) by the nasal route. CD4+ T-cell proliferation was assessed in cervical (CLN), iliac (ILN), mesenteric (MLN) lymph nodes and spleen (SPL) by CFSE dilution at different time points following immunization.(A) OVA-specific proliferation of OT-II CD4+ T cells was analysed in different lymphoid organs three days after immunization. Histograms are gated on CD4+CFSE+ population, with light scatter properties of lymphocytes. The number of generation is reported for each peak. (B) Graphs reporting the percentages of CD4+ CFSE+ T cells for every cell generation in CLN, ILN, MLN and SPL 0, 57, 72, 84 and 96 after immunization. Values are reported as mean ± standard error of the mean (SEM).

To characterize T cell clonal expansion and dissemination, ovalbumin (OVA)-specific transgenic CD4+ T cells, labeled with CFSE, were adoptively transferred into recipient mice. CFSE is a vital dye that can label cells very stably by covalently coupling to intracellular molecules, and it can be used to monitor lymphocyte proliferation due to the progressive halving of the dye fluorescence following cell division [33] (Fig 1A). The induction of OVA-specific CD4+ T cell clonal expansion was analyzed in draining cervical lymph nodes, distal iliac and mesenteric lymph nodes and spleen at 0, 57, 72, 84 and 96 hours after nasal immunization with ovalbumin OVA plus CpG ODN 1826 adjuvant (Fig 1B). The time course analysis of antigen-specific T-cell proliferation provided important information about the dynamic of the clonal expansion and the dissemination of antigen-specific primed CD4+ T cells. The starting time point was fixed at *T* = 72 hours, when it was assumed that the migration process of T cells from the draining lymph node starts. In fact, no proliferated T cells were detected in the distal lymph nodes and spleen until *T* = 84 hours (Fig 1B), with a very low frequency of transgenic CD4+ T cells in the generations one, two and three suggesting that proliferated T cells observed in these organs are migrated from draining lymph nodes, as previously reported ([34],[35]) (Fig 1B). The time step was estimated as Δ*t* = 4 hours considering that in draining cervical lymph nodes cells start to divide about 42 hours after nasal immunisation (data not shown) and that 4 new cell generations are observed 57 hours after immunisation (Fig 1B).

### Mathematical model of the network

Each component of the model is a node of the network. The draining lymph nodes are the source for our proliferation and trafficking model, while the distal lymph nodes and the spleen are sink nodes for T cells. The dissemination process is modeled through a suitable number of transfer nodes, depending on the relative magnitude of the transfer time from the source node to the sink nodes and the elementary time step of the dynamic evolution of the cell population. For example, if we have prior knowledge that the migration time from the source to the sinks is approximately 12 hours and we adopt a time step of 4 hours, we will simulate this setup by characterizing the transfer compartment (TR) through three transitions (see Section **A** in S1 Text). The set of transfer nodes represents the transfer compartment of the network, including lymphatic and blood vessels, which are assumed to be the main routes for T cells dissemination. In each node, T cells proliferate according to the basic proliferation process and the migration or immigration flows (see Section **A** in S1 Text).

In Section **A** of S1 Text, first and second order moments of cell counts in each model compartment are computed analytically. The basic proliferation model underlying the equations is a MGW process with migration and immigration. In our experimental setup, the model compartments are represented by the draining lymph nodes, which act as a source for the network, the transfer compartment, made by three serial stages, the spleen and the distal lymph nodes, which act as sinks for the network. Although we will explicitly mention these compartments, we point out that the presented results hold for any dissemination network with no feedback (recirculation) paths.

The basic proliferation process is assumed to follow a MGW branching process. This process assumes a given time step for the proliferation, during which each cell of a given generation has a certain probability of dividing, giving rise to two cells of the next generation, dying or remaining in quiescence. Although this setting is the simplest one in the theory of branching processes, the multi type nature of the process allows for characterizing and tracking different generations of T cells. This allows us to capture one of the complexity sources of the model, i.e., the time variation of cell propensity to division or death. In fact, the generation number of a cell is commonly considered as a meaningful indicator in the decision making process of a cell leading to division, apoptosis or differentiation ([14],[18],[31]). On the other hand, the simplicity of the probabilistic structure of the resulting Markov chain allows us to derive closed form expressions for first and second order moments of the process also in the presence of immigration and migration in/from certain nodes of the model (see Section **A** in S1 Text). Let us now mention a couple of aspects of *in vivo* CFSE data which impact over model statistical inference. First, we know that there are limitation on measurements in the transfer compartments, i.e., lymph and blood vessels. Hence, in our statistical identification scheme we will exploit only data from lymph nodes and spleen. A second issue regards the sampling of the proliferation process to collect CFSE data. To collect a sample of measurements requires sacrificing an animal at a chosen time point after immunization. In this case, performing measurements at different time points (or even repeating the measurements at a given time point) asks for sacrificing different immunized animals. This means that measurements taken at different time points refer to individuals which can be quite different. This situation makes any multi type mathematical model of cell population kinetics based on cell counts not suitable for reliable statistical inference [36]. To overcome this problem, we further elaborate the cell count model to generate a model involving relative frequencies of each type of cells, assuming as normalizing variable the total number of cells recorded at each time point. In doing this, starting from the method proposed in the seminal paper [36], we derive first and second order moments of different type cell relative frequencies. Beyond the above mentioned individual heterogeneity problem, this approach shows additional advantages, the most important being that it allows us to derive analytically a normal approximation of the log likelihood function of cell relative frequencies in the lymph nodes, whereas computation of such a function is intractable when dealing with cell counts [14]. Moreover, in the special case when only the draining lymph node is considered, it allows for analytic computation of the exact asymptotic likelihood function ([28],[36]).

### Parameter estimation and sensitivity analysis

In this subsection we report the numerical results obtained by performing the lymph node network model identification (see Section **B** in S1 Text for the model estimation procedure) on data recorded in the immunization experiments reported above. The definition of biological variables and model parameters are in Table 1.

**Table 1:** Network model parameter definition and biological variable measured. For the variables *Z*_*dr*,*i*_(*n*), *Z*_*il*,*i*_(*n*), *Z*_*mes*,*i*_(*n*) and *Z*_*spl*,*i*_(*n*) see also Section **A** in S1 Text.

Biological data	Definition	Units	Assay
*Z*_*dr*,*i*_(*n*)	Number of OT-II cells for each generation identified by CFSE dilution (*i*) in draining lymph nodes	Cells per generation	Flow Cytometry
*Z*_*il*,*i*_(*n*)	Number of OT-II cells for each generation *i* identified by CFSE dilution in iliac lymph node	Cells per generation	Flow Cytometry
*Z*_*mes*,*i*_(*n*)	Number of OT-II cells for each generation *i* identified by CFSE dilution in mesenteric lymph nodes	Cells per generation	Flow cytometry
*Z*_*spl*,*i*_(*n*)	Number of OT-II cells for each generation *i* identified by CFSE dilution in the spleen	Cells per generation	Flow cytometry
*γ*_*i*_	Probability of division for T cells in generations *i*	adimensional	Estimated
*δ*_*i*_	Probability of quiescence for T cells in generations *i*	adimensional	Estimated
*m*_*i*_	Probability of migration for T cells in generation *i*	adimensional	Estimated
*ρ*_*il*_	Probability of splitting towards iliac lymph nodes	adimensional	Estimated
*ρ*_*mes*_	Probability of splitting towards mesenteric lymph nodes	adimensional	Estimated
*ρ*_*spl*_	Probability of splitting towards spleen	adimensional	Estimated
Δ*t*	Probabilistic time of division, quiescence, death and migration	Hours	Estimated

According to the experimental results in [29], we assumed different division and quiescence probability parameters *γ*_*i*_, *δ*_*i*_, for the generations *i* = 0,1,2, while the probability parameters for generations *i* > 2 were assumed equal to those for *i* = 2. Moreover, we assumed that the proliferation parameters *δ*_*i*_, *γ*_*i*_, are equal for all the model compartments. The overall parameter vector ***θ*** contains the division and quiescence probabilities for the various cell generations *δ*_*i*_, *γ*_*i*_, *i* = 0,1,2, the migration probability *m* from the draining lymph node (here we assume that migration probabilities of the various generations are equal, except for the generations 0 and *p* which do not migrate) and the splitting probabilities *ρ*_*spl*_, *ρ*_*il*_, *ρ*_*mes*_ of migrating cells in the network sink nodes [37], (again we assume that splitting probabilities of the various generations are equal) (see Table 2). In order to estimate the model parameter vector ***θ***, we need to know the T cell counts at the initial time point in the draining lymph node, which is the network source. The starting time point for the counting process (*n* = 0) has been fixed at *T* = 72 hours as stated in Subsection CD4+ T cell proliferation experimental data.

**Table 2:** Model network parameters assumptions.

**Parameter**	**Description**
Time step	The time step of the model was fixed as Δ*t* = 4 hours. It was estimated considering 42 hours after nasal immunisation as the average time in which a cell starts to divide and the 4 new cell generations observed in draining cervical lymph nodes 57 hours after immunisation.
Division and quiescence probabilties	Different division and quiescence probability parameters *γ*_*i*_, *δ*_*i*_ were assumed for the generations *i* = 0,1,2, while the probability parameters for generations *i* > 2 were assumed equal to those for *i* = 2, as shown in [29].
Migration probabilities	It was assumed that the cells in generation *i* = 1,2,3 migrate and their migration probabilities are equal.
Immigration probabilities	It was assumed that immigration probabilities of the various generations towards distal lymph nodes are equal except for the generations *i* = 0 which does not migrate.

**Fig 2:**
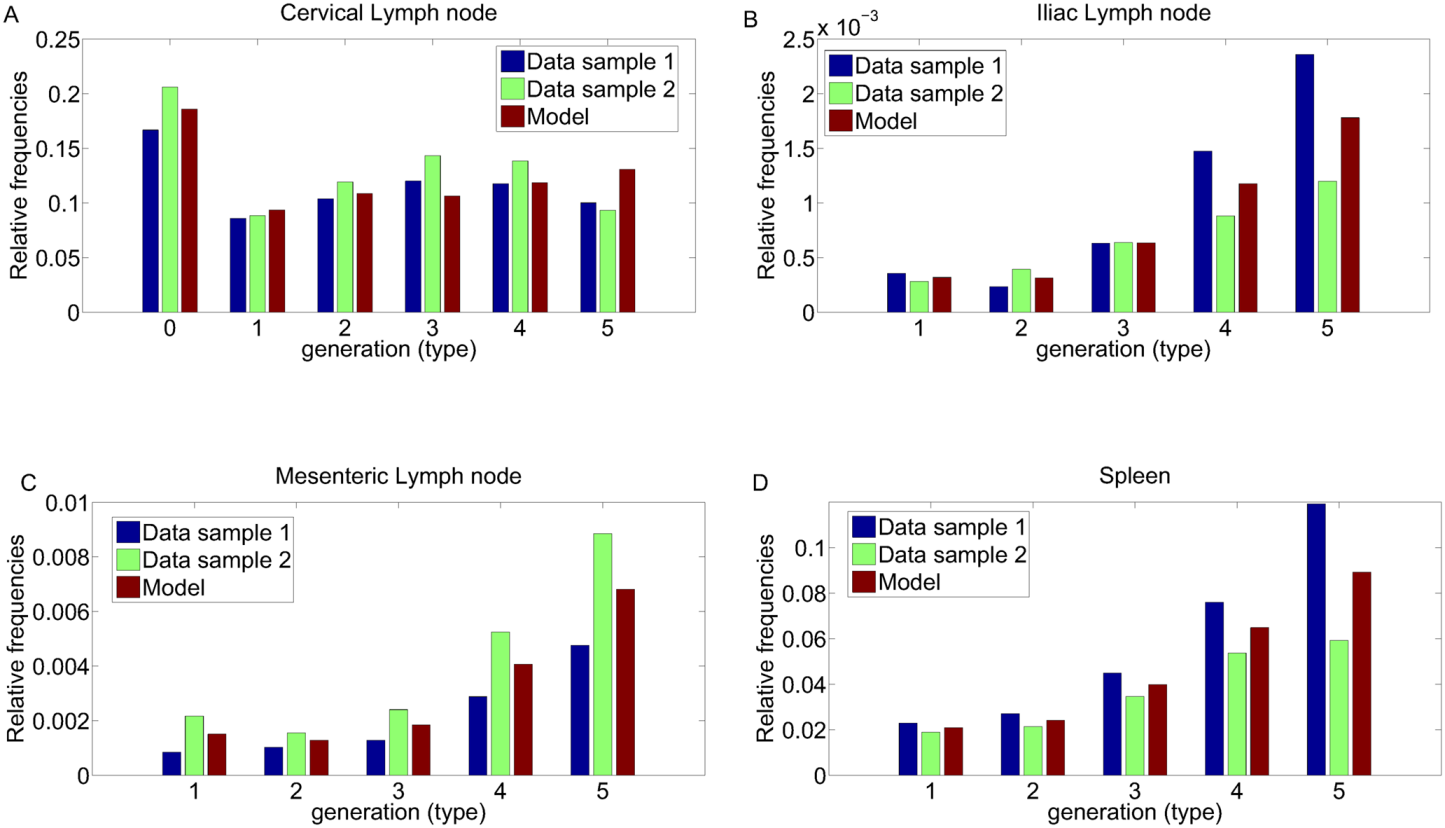
Model fitting. Relative frequencies fitting at time point *n* = 6 (96 hours) post immunization for all the T cell generations.(A) Draining lymph nodes (B) Iliac lymph nodes (C) Mesenteric lymph nodes (D) Spleen. The histograms indicate the relative frequencies values predicted by the model compared to relative frequencies data obtained by *in vivo* animal experiment. Data samples from two animals representative of a group of five are reported.

Table 3 shows parameter estimates and standard errors obtained through minimization of the normal approximation of the negative log likelihood function of the cell frequencies (see Section **B** of S1 Text). The standard errors are computed with the Fisher information matrix. Fig 2 shows the good quality fitting of the relative frequencies predicted by the model to the experimental data from the experiments in all the model compartments at time point *n* = 6 (*T* = 96 hours). At this time point, in the spleen and in draining lymph nodes about 1.7 × 10^5^ and 1.5 × 10^5^ antigen-specific CD4+ T cells/organ were observed, respectively; in distal iliac and mesenteric lymph nodes about 5 × 10^3^ and 2 × 10^4^ cells/organ were detected, respectively (data not shown).

**Table 3:** Network model parameter estimates and standard errors.

Parameter	Notation	Value	Std. Err.
Probability of quiescence for T cells in generation 0	*δ*_0_	0.43	0.05
Probability of division for T cells in generation 0	*γ*_0_	0.06	0.01
Probability of quiescence for T cells in generation 1	*δ*_1_	0.31	0.05
Probability of division for T cells in generation 1	*γ*_1_	0.29	0.014
Probability of quiescence for T cells in generation 2	*δ*_2_	0.23	0.012
Probability of division for T cells in generation 2	*γ*_2_	0.24	0.09
Probability of migration for T cells in generations 1, 2, 3	*m*	0.14	0.013
Probability of splitting towards Spleen	*ρ*_*spl*_	0.95	0.017
Probability of splitting towards iliac lymph nodes	*ρ*_*il*_	0.01	0.005
Probability of splitting towards mesenteric lymph nodes	*ρ*_*mes*_	0.04	0.0201

**Fig 3:**
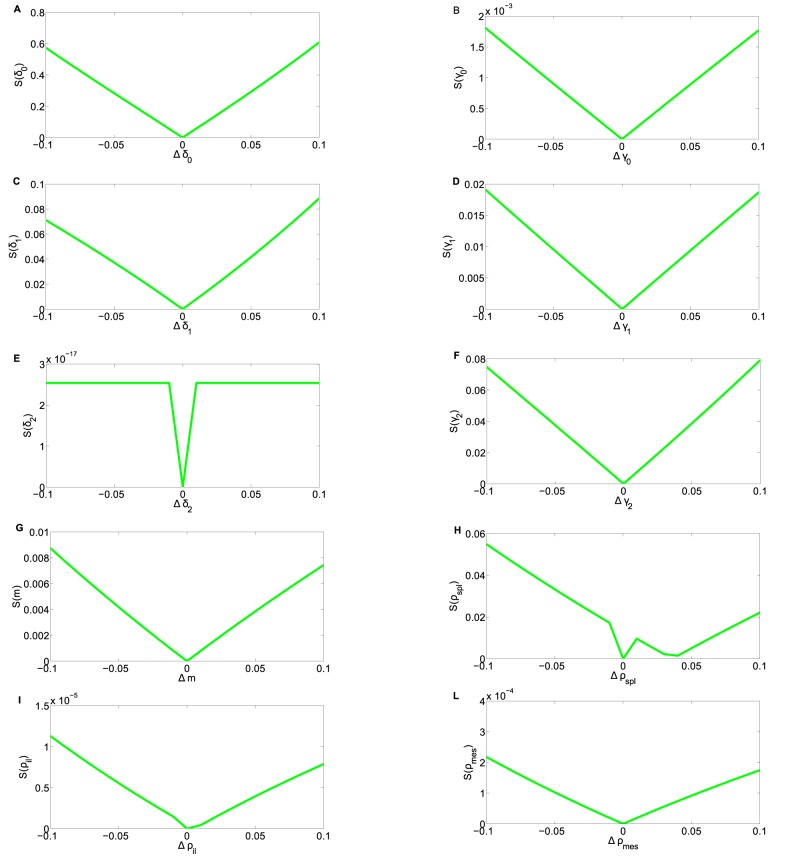
Sensitivities coefficients of model parameters. We computed the sensitivity coefficient *S*(*θ*_*i*_) for proliferation, migration and splitting parameters for $\Delta \theta_{i}\in [-0.1{\hat{\theta }}_{i},0.1{\hat{\theta }}_{i}],i=0,1,\ldots ,10$.(A)*δ*_0_: Probability of quiescence for *naive* T cell;(B) *γ*_0_: Probability of proliferation for *naive* T cell;(C)*δ*_1_: Probability of quiescence for T cells in generation 1;(D)*γ*_1_: Probability of proliferation for T cells in generation 1;(E)*δ*_2_: Probability of quiescence for T cells in generation 2; (F)*γ*_2_: Probability of proliferation for T cells in generation 2; (G)*m*: Probability of migration; (H)*ρ*_*spl*_: Probability of splitting towards spleen; (I)*ρ*_*il*_: Probability of splitting towards iliac limph node; (L)*ρ*_*mes*_: Probability of splitting towards mesenteric lymph node.

Some comments are in order when examining Table 3. First of all, we point out that in spite of the limited number of measurement time points (*n* = 0,3,6), the magnitude of standard errors indicate a good quality of the parameter estimates. This is due to the fact that spatially distributed measurements are informative for model parameter evaluation. Actually, from the information viewpoint, measurements in different points of the network are equivalent to repeated measurements in the same site. To further check reliability and significance of the estimates, a numerical sensitivity analysis of the estimates has been carried out. This analysis quantifies the extent to which each parameter affects the relative frequencies predicted by the model estimated over the experimental data available. In this sense, it provides a quantitative evaluation of the potential of each parameter in explaining the experimental data. To this purpose, we introduce the following definition of relative sensitivity *S*(*θ*_*i*_):

$$\begin{array}{c} S\left(\theta_{i}\right)=\frac{|\Delta 𝓛\left(\hat{\theta }\right)|/|𝓛\left(\hat{\theta }\right)|}{|\Delta \theta_{i}|/{\hat{\theta }}_{i}},\;i=1,\ldots ,10\;, \end{array}$$

where $\hat{\text{θ}}={\left[{\hat{\theta }}_{1},\ldots ,{\hat{\theta }}_{10}\right]}^{T}$ is the parameter estimate, $𝓛(\hat{\text{θ}})$ is the optimized cost function at $\text{θ}=\hat{\text{θ}}$, $\Delta \theta_{i}\in [-0.1{\hat{\theta }}_{i},0.1{\hat{\theta }}_{i}]$ and

$$\begin{array}{c} \Delta 𝓛\left(\hat{\theta }\right)=𝓛\left(\hat{\theta }+\Delta \theta_{i}\;{\text{e}}_{i}\right)-𝓛\left(\hat{\theta }\right), \end{array}$$

where **e_i_** is a vector with null components, except for the *i* – *th* entry which is 1. Fig 3 shows the relative sensitivity functions computed for each of the parameters. We notice that sensitivities of parameters *δ*_0_, *δ*_1_, *ρ*_*spl*_, *γ*_2_ are the largest ones, showing that they represent the parameters which capture most information contained in the experimental data sets.

The estimate numerical values in Table 3, showed that cells that lost the competition for the antigen and are still undivided 3 days after immunization has a negligible probability to divide and do not contribute any longer to the proliferation process Rather, the number of *naive* cells decreases exponentially with a mortality rate greater than 0.5: this agrees nicely with the biological knowledge on the immune response evolution. The migration rate from the draining lymph node is around 14%, while the splitting rate among the distal lymphoid organs shows a clear prevalence of the migration towards the spleen.

**Fig 4:**
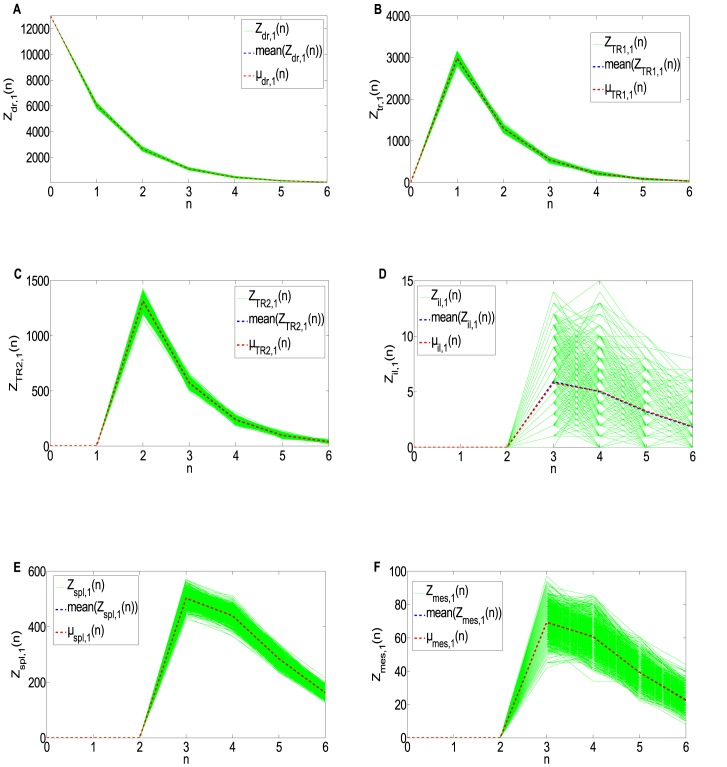
Stochastic simulations of T cells in generation 1 in the model compartments. X-axis: timepoint (n). Y-axis: random realizations of T cells in generation 1. The green lines represent the 1000 simulated sample paths. The red dashed line indicates the expected value predicted by the model and the blue dashed line represents the mean value of the sample paths obtained through stochastic simulations. The T cells in generation 1 simulations show the typical T cell clonal expansion and contraction profiles. (A) Draining Lymph node; (B)Migrating CD4+ T cell in the transfer compartment; (C) TR compartment; (D) Iliac lymph node compartment; (E) Spleen compartment; (F) Mesenteric lymph node compartment.

As a final test on the model, we simulated 1000 stochastic sample paths using the parameter estimates. Fig 4 shows the simulated paths of the proliferation process for T cells of first generation in the various model compartments.

## Discussion

T cell priming is an essential event for the induction of the primary immune response to vaccination. It is influenced by the type of vaccine formulation (antigen, adjuvant, delivery system), the dose and the route of administration. The characterization of T cell priming is therefore critical in order to develop optimal prime-boost combinations capable of eliciting the type of immune response required to contrast a specific pathogen [3]. To this purpose, quantification of the adaptive immune response temporal dynamics through the features of T cell proliferation and dissemination represents a key challenge for experimental immunologists and vaccinologists.

The application of systems biology in the context of vaccinology has recently been proposed as a new powerful tool to model and characterize host immune responses to vaccination [38] and offers great promise for future translation of basic immunology research advances into successful vaccines [39]. System immunology provides indeed new tools to assess immune responses investigating the cell dynamics following immunisation, understanding the mechanisms of cell activation and deriving models of efficient vaccine induced immune responses [39]. Furthermore mathematical models represent an attractive tool to gain insight into the complexity of the immune response by providing quantitative information on the rates of division, death and migration of T cells upon vaccination and to understand the effectiveness of different vaccination strategies through computational simulation. While mathematical models have been widely applied to *in vitro* studies, *in vivo* analysis raises several difficulties, mainly due to the fact that a lymph node is not an isolated site but is part of the complex immunological system. In the present work we succeeded in constructing a stochastic model for the proliferation and dissemination of antigen-specific CD4+ T cells upon vaccination. The proliferation mechanism is based on the probabilistic laws typical of multi-type Galton Watson branching processes. In this context, a further contribution of the paper consists in the closed form derivation of a normal approximation of the log likelihood function of relative frequencies in the different nodes of the dissemination network. This allows to deal with cell count data coming from *in vivo* experiments where measurements at different time points are taken on different individuals.

Since in our experimental data, the intranasal immunization route is adopted, we consider as main compartments of the network, the cervical lymph nodes as draining compartment and the spleen, iliac and mesenteric lymph nodes as distal compartments. We identified this model on the basis of CFSE data in mice, obtaining key immunological parameters at generation level, which could be difficult to retrieve only through CFSE histograms.

Model parameter estimates reveal several features of the antigen-specific CD4+T cells dynamics. While we have previously characterised the proliferation dynamic during the early phase of T cell priming (from 42 to 57 hours after immunization) only in the draining lymph nodes ([28],[29]), the results in this work characterize the later phase (from 72 to 96 hours after immunization) and dissemination in the entire lymphoid network. Interestingly, the proliferation during the time period from 72 to 96 hours is slower than during the first phase of T cell priming, showing that the time of cell division is faster in the early phase, as previously observed also for CD8+ T cells [40].

Furthermore, the estimated parameters showed that *naive* T cells have a negligible probability to enter in division at late time points such as 96 hours after immunization, and their number decreases exponentially with a mortality rate greater than 0.5.

This phenomenon could be due to the lack of OVA- pulsed antigen presenting cells at this time point, that leads to an increased mortality of *naive* T cells in the absence of an appropriate stimulus. In fact, it was previously demonstrated that after nasal immunization with a fluorescent OVA plus CpG ODN 1826 antigen bearing, dendritic cells were detected in draining lymph nodes only within 72 hours [28]. As a further consideration, we observe that T cell migration from the draining lymph nodes to the blood is one of the most meaningful events in the time period from 72 to 96 hours which activates dissemination in the network after immunization. With reference to the splitting parameters, the obtained values show that the spleen represents the largest storage of CD4+ T cells. This result is in line with the conclusions of [30] which developed a mathematical model indicating that the spleen represents the main source of effector CD8+ T cells against viral infections and for T cell memory production.

By looking at the stochastic simulations shown in Fig 4, we observe the typical T cell dynamics pathways, i.e, clonal expansion after antigen encounter and the subsequent contraction due to the antigen clearance and cell apoptosis, leaving only a small pool of memory T cells. In particular, T cells in generation 1 in the draining lymph nodes only exhibit the decaying phase, due to the combination of high death rate, high migration in the blood and lymphatic vessels and low propensity to division of *naive* T cells.

In conclusion, these results provide a complete quantitative explanation of CD4+ T cells *in vivo* pathways following nasal immunization, paving the way for future applications of this model to other immune cells, infections or vaccination strategies. Finally, we highlight that since the model is based on the general theory of branching processes, it can be applied in a broader range of biological modeling problems where proliferation and dissemination in a network are of interest. Specifically, the application of this model to the study of immune response upon immunization is highly relevant to predict vaccine immunogenicity and for the rational development of appropriate prime-boost vaccine strategies.

## Supporting Information

S1 Text Statistical moments of the stochastic branching process model network. (PDF)

## References

[1] Ciabattini A, Pettini E, Andersen P, Pozzi G, Medaglini D. Primary activation of antigen-specific naive CD4+ and CD8+ T cells following intranasal vaccination with recombinant bacteria. Infection and immunity. 2008;76(12):5817–5825. 10.1128/IAI.00793-08 18838521 PMC2583588

[2] Ciabattini A, Pettini E, Arsenijevic S, Pozzi G, Medaglini D. Intranasal immunization with vaccine vector Streptococcus gordonii elicits primed CD4+ and CD8+ T cells in the genital and intestinal tracts. Vaccine. 2010;28(5):1226–1233. 10.1016/j.vaccine.2009.11.021 19945415

[3] Ciabattini A, Pettini E, Medaglini D. CD4+ T Cell Priming as Biomarker to Study Immune Response to Preventive Vaccines. Frontiers in Immunology. 2013;4 10.3389/fimmu.2013.00421 PMC3850413 24363656

[4] McHeyzer-Williams LJ, Pelletier N, Mark L, Fazilleau N, McHeyzer-Williams MG. Follicular helper T cells as cognate regulators of B cell immunity. Current Opinion in Immunology. 2009;21(3):266–273. 10.1016/j.coi.2009.05.010 19502021 PMC2731669

[5] Galli G, Medini D, Borgogni E, Zedda L, Bardelli M, Malzone C, et al Adjuvanted H5N1 vaccine induces early CD4+ T cell response that predicts long-term persistence of protective antibody levels. Proceedings of the National Academy of Sciences. 2009;106(10):3877–3882. 10.1073/pnas.0813390106 PMC2646626 19237568

[6] Spensieri F, Borgogni E, Zedda L, Bardelli M, Buricchi F, Volpini G, et al Human circulating influenza-CD4+ ICOS1+ IL-21+ T cells expand after vaccination, exert helper function, and predict antibody responses. Proceedings of the National Academy of Sciences. 2013;110(35):14330–14335. 10.1073/pnas.1311998110 PMC3761599 23940329

[7] Fiorino F, Pettini E, Pozzi G, Medaglini D, Ciabattini A. Prime-boost strategies in mucosal immunization affect local IgA production and the type of Th response. Frontiers in immunology. 2013;4 10.3389/fimmu.2013.00128 23755051 PMC3665932

[8] Dereuddre-Bosquet N, Baron ML, Contreras V, Gosse L, Mangeot I, Martinon F, et al HIV specific responses induced in nonhuman primates with ANRS HIV-Lipo-5 vaccine combined with rMVA-HIV prime or boost immunizations. Vaccine. 2015;33(20):2354–2359. 10.1016/j.vaccine.2015.03.032 25839103

[9] McKay PF, Cope AV, Mann JF, Joseph S, Esteban M, Tatoud R, et al Glucopyranosyl lipid A adjuvant significantly enhances HIV specific T and B cell responses elicited by a DNA-MVA-protein vaccine regimen. PloS one. 2014;9(1):e84707 10.1371/journal.pone.0084707 24465426 PMC3900398

[10] De Boer RJ, Perelson AS. Estimating division and death rates from CFSE data. Journal of Computational and Applied Mathematics. 2005;184(1):140–164. 10.1016/j.cam.2004.08.020

[11] Hawkins ED, Turner ML, Dowling MR, Van Gend C, Hodgkin PD. A model of immune regulation as a consequence of randomized lymphocyte division and death times. Proceedings of the National Academy of Sciences. 2007;104(12):5032–5037. 10.1073/pnas.0700026104 PMC1821128 17360353

[12] De Boer RJ, Perelson AS. Quantifying T lymphocyte turnover. Journal of Theoretical Biology. 2013;327:45–87. 10.1016/j.jtbi.2012.12.025 23313150 PMC3640348

[13] Luzyanina T, Roose D, Bocharov G. Distributed parameter identification for a label-structured cell population dynamics model using CFSE histogram time-series data. Journal of Mathematical Biology. 2009;59(5):581–603. 10.1007/s00285-008-0244-5 19096849

[14] Yates A, Chan C, Strid J, Moon S, Callard R, George AJT, et al Reconstruction of cell population dynamics using CFSE. BMC bioinformatics. 2007;8(1):196 10.1186/1471-2105-8-196 17565685 PMC1929124

[15] Hyrien O, Chen R, Zand MS. An age-dependent branching process model for the analysis of CFSE-labeling experiments. Biology Direct. 2010;. 10.1186/1745-6150-5-41 20569476 PMC2914727

[16] Bains I, Antia R, Callard R, Yates AJ. Quantifying the development of the peripheral naive CD4+ T-cell pool in humans. Blood. 2009;113(22):5480–5487. 10.1182/blood-2008-10-184184 19179300 PMC2689049

[17] Miao H, Jin X, Perelson AS, Wu H. Evaluation of multitype mathematical models for CFSE-labeling experiment data. Bulletin of Mathematical Biology. 2012;74(2):300–326. 10.1007/s11538-011-9668-y 21681605 PMC3196768

[18] Duffy KR, Hodgkin PD. Intracellular competition for fates in the immune system. Trends in Cell Biology. 2012;22(9):457–464. 10.1016/j.tcb.2012.05.004 22727035

[19] Banks HT, Clayton Thompson W. Mathematical models of dividing cell populations: Application to CFSE data. Mathematical Modelling of Natural Phenomena. 2012;7(05):24–52. 10.1051/mmnp/20127504

[20] Hasenauer J, Schittler D, Allgöwer F. Analysis and Simulation of Division-and Label-Structured Population Models. Bulletin of Mathematical Biology. 2012;74(11):2692–2732. 23086287 10.1007/s11538-012-9774-5

[21] Thomas-Vaslin V, Altes HK, de Boer RJ, Klatzmann D. Comprehensive assessment and mathematical modeling of T cell population dynamics and homeostasis. The Journal of Immunology. 2008;180(4):2240–2250. 10.4049/jimmunol.180.4.2240 18250431

[22] Stekel DJ, Parker CE, Nowak MA. A model of lymphocyte recirculation. Immunology Today. 1997;18(5):216–221. 10.1016/S0167-5699(97)01036-0 9153952

[23] Antia R, Ganusov VV, Ahmed R. The role of models in understanding CD8+ T-cell memory. Nature Reviews Immunology. 2005;5(2):101–111. 10.1038/nri1550 15662368

[24] Sallusto F, Lanzavecchia A, Araki K, Ahmed R. From vaccines to memory and back. Immunity. 2010;33(4):451–463. 10.1016/j.immuni.2010.10.008 21029957 PMC3760154

[25] Asquith B, Debacq C, Florins A, Gillet N, Sanchez-Alcaraz T, Mosley A, et al Quantifying lymphocyte kinetics in vivo using carboxyfluorescein diacetate succinimidyl ester. Proceedings of the Royal Society B: Biological Sciences. 2006;273(1590):1165–1171. 10.1098/rspb.2005.3432 16600897 PMC1560268

[26] Ganusov VV, Pilyugin SS, De Boer RJ, Murali-Krishna K, Ahmed R, Antia R. Quantifying cell turnover using CFSE data. Journal of Immunological Methods. 2005;298(1):183–200. 10.1016/j.jim.2005.01.011 15847808

[27] Banks HT, Sutton KL, Thompson WC, Bocharov G, Roose D, Schenkel T, et al Estimation of cell proliferation dynamics using CFSE data. Bulletin of Mathematical Biology. 2011;73(1):116–150. 10.1007/s11538-010-9524-5 20195910 PMC2911498

[28] Pettini E, Prota G, Ciabattini A, Boianelli A, Fiorino F, Pozzi G, et al Vaginal Immunization to Elicit Primary T-Cell Activation and Dissemination. PloS One. 2013;8(12):e80545 10.1371/journal.pone.0080545 24349003 PMC3857820

[29] Boianelli A, Pettini E, Prota G, Medaglini D, Vicino A. Identification of a branching process model for adaptive immune response. In: Proc. IEEE 52nd Conference on Decision and Control (CDC), 2013. IEEE; 2013. p. 7205–7210.

[30] Wu H, Kumar A, Miao H, Holden-Wiltse J, Mosmann TR, Livingstone AM, et al Modeling of influenza-specific CD8+ T cells during the primary response indicates that the spleen is a major source of effectors. The Journal of Immunology. 2011;187(9):4474–4482. 10.4049/jimmunol.1101443 21948988 PMC3197949

[31] Schlub TE, Venturi V, Kedzierska K, Wellard C, Doherty PC, Turner SJ, et al Division-linked differentiation can account for CD8+ T-cell phenotype in vivo. European Journal of Immunology. 2009;39(1):67–77. 10.1002/eji.200838554 19130548

[32] Pape KA, Kearney ER, Khoruts A, Mondino A, Merica R, Chen ZM, et al Use of adoptive transfer of T-cell antigen-receptor-transgenic T cells for the study of T-cell activation in vivo. Immunological Reviews. 1997;156(1):67–78. 10.1111/j.1600-065X.1997.tb00959.x 9176700

[33] Pettini E, Ciabattini A, Pozzi G, Medaglini D. Adoptive transfer of transgenic T cells to study mucosal adjuvants. Methods. 2009;49(4):340–345. 10.1016/j.ymeth.2009.03.026 19409994

[34] Ciabattini A, Pettini E, Fiorino F, Prota G, Pozzi G, Medaglini D. Distribution of primed T cells and antigen-loaded antigen presenting cells following intranasal immunization in mice. PloS One. 2011;6(4):e19346 10.1371/journal.pone.0019346 21559409 PMC3084830

[35] Medaglini D, Ciabattini A, Cuppone A, Costa C, Ricci S, Costalonga M, et al In vivo activation of naive CD4+ T cells in nasal mucosa-associated lymphoid tissue following intranasal immunization with recombinant Streptococcus gordonii. Infection and Immunity. 2006;74(5):2760–2766. 10.1128/IAI.74.5.2760-2766.2006 16622213 PMC1459748

[36] Yakovlev AY, Yanev NM. Relative frequencies in multitype branching processes. The Annals of Applied Probability. 2009;p. 1–14. 10.1214/08-AAP539

[37] Mandl JN, Liou R, Klauschen F, Vrisekoop N, Monteiro JP, Yates AJ, et al Quantification of lymph node transit times reveals differences in antigen surveillance strategies of naive CD4+ and CD8+ T cells. Proceedings of the National Academy of Sciences. 2012;109(44):18036–18041. 10.1073/pnas.1211717109 PMC3497782 23071319

[38] Pulendran B, Li S, Nakaya HI. Systems vaccinology. Immunity. 2010;33(4):516–529. 10.1016/j.immuni.2010.10.006 21029962 PMC3001343

[39] Six A, Bellier B, Thomas-Vaslin V, Klatzmann D. Systems biology in vaccine design. Microbial biotechnology. 2012;5(2):295–304. 10.1111/j.1751-7915.2011.00321.x 22189033 PMC3815789

[40] Lawrence CW, Braciale TJ. Activation, differentiation, and migration of naive virus-specific CD8+ T cells during pulmonary influenza virus infection. The Journal of Immunology. 2004;173(2):1209–1218. 10.4049/jimmunol.173.2.1209 15240712

