# Supplementary material for: A Stochastic Model for CD4+ T Cell Proliferation and Dissemination Network in Primary Immune Response
Source: PLoS One. 2015 Aug 24;10(8):e0135787. doi: 10.1371/journal.pone.0135787 (PMC4547705; doi:10.1371/journal.pone.0135787)
Supplement: S1 Text — (PDF) [file pone.0135787.s001.pdf]

# A Stochastic Model for CD4+ T cell Proliferation and Dissemination Network in Primary Immune Response

# Supporting Information

Alessandro Boianelli[1], Elena Pettini[2], Gennaro Prota[2], Donata Medaglini[2], Antonio Vicino[3]

[1] Systems Medicine of Infectious Diseases Group and Braunschweig Integrated Centre of Systems Biology, Department of Systems Immunology, Helmholtz Centre for Infection Research, Inhoffenstrasse 7, Braunschweig, Germany.

[2] Laboratorio di Microbiologia Molecolare e Biotecnologie, Dipartimento di Biotecnologie Mediche,Università di Siena, Viale Bracci 1, 53100 Siena, Italy.

[3]Dipartimento di Ingegneria dell'Informazione e Science Matematiche, Università di Siena, Via Roma 56 , 53100 Siena, Italy.

## Matherial and Methods

## A. Moments for the network compartments

In this Section we provide explicit expressions for the first and second order moments of cell counts in the different compartments of the network model. The interested reader is referred to (*41*) for the derivation of the formulas.

### Draining lymph node

The generation of a cell, which is defined as the number division steps the cells undergoes before its birth, represents the type of that cell. We denote by $\Delta t$ the sampling time (or time step) of the discrete time process and by $n$ the generic time point $t = n\ \Delta t$. Let the state of the population in the draining lymph node at time $n$ be represented by the vector $\mathbf{Z}_{dr}(n) = [Z_{dr,0}(n), Z_{dr,1}(n), Z_{dr,2}(n), \ldots, Z_{dr,p}(n)]$, where $Z_{dr,i}(n)$ are random

variables representing the counts of cells after $i$ divisions ($p$ is the maximum generation considered).

The transition matrix $\tilde{\mathbf{M}}_{dr} \in \mathbb{R}^{(p+1,p+1)}$ which maps cell counts at time $n$ to cell counts at time $n+1$ is given by ( *42* ):

$$\tilde{\mathbf{M}}_{dr} = \begin{pmatrix} \delta_0 & 2\gamma_0 & 0 & 0 & 0 & 0 \\ 0 & \delta_1 & 2\gamma_1 & 0 & \dots & \dots \\ 0 & 0 & \delta_2 & 2\gamma_2 & 0 & 0 \\ \vdots & \vdots & 0 & \ddots & \ddots & 0 \\ \vdots & \vdots & \vdots & 0 & \delta_{p-1} & 2\gamma_{p-1} \\ 0 & 0 & 0 & 0 & 0 & \delta_p \end{pmatrix} . \quad (1)$$

The mean value $\boldsymbol{\mu}_{dr}$ is given by:

$$\boldsymbol{\mu}_{dr}(n) = [\mu_{dr,0}(n), \dots, \mu_{dr,p}(n)] = E[\mathbf{Z}_{dr}(n)] = \boldsymbol{\mu}_{dr}(n-1)\tilde{\mathbf{M}}_{dr} . \quad (2)$$

To compute the covariance matrix of cell counts, we introduce the matrix $\mathbf{V}_l$ representing the one step covariance matrix for one cell present in the state $\mathbf{Z}(0) = \mathbf{e}_l$, being $\mathbf{e}_l, 0 \leq l \leq p$ a vector whose $l+1-th$ component is 1 and whose other components are 0 (see (*42*)). Then, the covariance matrix $\mathbf{S}_{dr}(n)$ turns out to be the sum of two terms, the first one accounting for the transition form $n-1$ to $n$ and the second one quantifying the intrinsic one step covariance increment due to the presence of cells of the various generations at time $n-1$:

$$\mathbf{S}_{dr}(n) = \tilde{\mathbf{M}}_{dr}^T \mathbf{S}_{dr}(n-1)\tilde{\mathbf{M}}_{dr} + \sum_{l=0}^{p} \mathbf{V}_l \mu_{dr,l}(n-1) . \quad (3)$$

Now, we extend this basic model by allowing the presence of migration from the draining lymph node. To this purpose, we introduce the r.v.'s $\eta_i$ $i = 1, 2, \dots, p-1$ representing binomial random variables associated to the cells of different types. We assume that $\eta_i = 1$ if the cell of type $i$ migrates in a time step $\Delta t$ and $\eta_i = 0$ if the cell of type $i$ doesn't migrate. We denote by $m_i$ and $1 - m_i,\ i = 1, 2, \dots, p-1$ the probabilities of the two possible events, respectively. Notice that, we assume here that the *naive* T cells (type 0) and the highest generation cells(type $p$) have null migration probabilities, i.e., $m_0 = m_p = 0$. This is in accordance with biological knowledge on the process and the fact that the CFSE measuring equipment has limited resolution. The transition matrix of the process in our source node $\mathbf{M}_{dr} \in \mathbb{R}^{p+1,p+1}$ in the presence of migration becomes:

$$\mathbf{M}_{dr} = \begin{pmatrix} \delta_0 & 2\gamma_0(1-m_1) & 0 & 0 & 0 & 0 \\ 0 & \delta_1(1-m_1) & 2\gamma_1(1-m_2) & 0 & \dots & \dots \\ 0 & 0 & \delta_2(1-m_2) & 2\gamma_2(1-m_3) & 0 & 0 \\ \vdots & \vdots & \vdots & \ddots & \ddots & 0 \\ \vdots & \vdots & \vdots & 0 & (1-m_{p-1})\delta_{p-1} & 2\gamma_{p-1} \\ 0 & 0 & 0 & 0 & 0 & \delta_p \end{pmatrix} . \tag{4}$$

With a slight abuse of notation, we will replace the mean value and covariance recursive equations (2) and (3) with the following expressions holding in the presence of migration:

$$\boldsymbol{\mu}_{dr}(n) = \boldsymbol{\mu}_{dr}(0)\mathbf{M}_{dr}^n \ , \tag{5}$$

$$\mathbf{S}_{dr}(n) = \mathbf{M}_{dr}^T \mathbf{S}_{dr}(n-1)\mathbf{M}_{dr} + \sum_{l=0}^{p} \mathbf{V}_l \mu_{dr,l}(n-1) \ , \tag{6}$$

where the one time step covariance matrices $V_l$ have the same meaning as before.

By introducing the migrating cell transition matrix $\mathbf{M}_{mig} \in \mathbb{R}^{(p+1,p+1)}$:

$$\mathbf{M}_{mig} = \begin{pmatrix} 0 & 2\gamma_0 m_1 & 0 & 0 & 0 & 0 \\ 0 & m_1\delta_1 & 2\gamma_1 m_2 & 0 & \dots & \dots \\ 0 & 0 & m_2\delta_2 & 2\gamma_2 m_3 & 0 & 0 \\ \vdots & 0 & \vdots & \ddots & \ddots & 0 \\ \vdots & \vdots & \vdots & 0 & m_{p-1}\delta_{p-1} & 0 \\ 0 & 0 & 0 & 0 & 0 & 0 \end{pmatrix} . \tag{7}$$

we obtain the expected value $\boldsymbol{\mu}_{mig}(n) \in \mathbb{R}^{p+1}$ and related covariance matrix $\mathbf{S}_{mig}(n) \in \mathbb{R}^{(p+1,p+1)}$ of migrating cells:

$$\boldsymbol{\mu}_{mig}(n) = \boldsymbol{\mu}_{dr}(n-1)\mathbf{M}_{mig} \ , \tag{8}$$

$$\mathbf{S}_{mig}(n)\mathbf{M}_{mig}^T \mathbf{S}_{dr}(n-1)\mathbf{M}_{mig} + \sum_{l=0}^{p} \mathbf{V}_{mig,l} \mu_{dr,l}(n-1) \ , \tag{9}$$

where $\mathbf{V}_{mig,l}$ represents the one step covariance matrix of one cell of type $l$ available for migration from the draining lymph node.

## Transfer compartment

In our model, as experimental tests showing that in our setup T cells take approximately 12 hours to move from the draining lymph node to the distal compartments, the transfer compartment is made up by 3 subcompartments named TR1, TR2, TR3. Of course, any different choice would not impact on the structure of the model, which is very flexible in this respect. Clearly, a rough knowledge of the migration time and of the time step, i.e., the mean division time, is necessary for appropriately structuring the transfer compartment.

We assume that migrating T cells $\mathbf{Z}_{mig}(n)$ at time point $n$ are located in the TR1 subcompartment. We denote by $\mathbf{Z}_{TR1}(n)$ the T cells counts vector of the first subcompartment. Then, the mean value $\boldsymbol{\mu}_{TR1}(n) \in \mathbb{R}^{p+1}$ and covariance matrix $\mathbf{S}_{TR1}(n) \in \mathbb{R}^{(p+1,p+1)}$ are:

$$\begin{aligned} \boldsymbol{\mu}_{TR1}(n) &= E[\mathbf{Z}_{TR1}(n)] = \boldsymbol{\mu}_{mig}(n) && (10) \\ \mathbf{S}_{TR1}(n) &= \mathbf{S}_{mig}(n) \ . && (11) \end{aligned}$$

After one time step in the TR1 compartment, the cells $\mathbf{Z}_{TR1}(n)$ undergo a transition while moving to the TR2 subcompartment. The transition matrix $\mathbf{M}_{tr} \in \mathbb{R}^{(p+1,p+1)}$ can be easily derived as

$$\mathbf{M}_{tr} = \begin{pmatrix} 0 & 0 & 0 & 0 & 0 & 0 \\ 0 & \delta_1 & 2\gamma_1 & 0 & \dots & \dots \\ 0 & 0 & \delta_2 & 2\gamma_2 & 0 & 0 \\ \vdots & 0 & \vdots & \ddots & \ddots & 0 \\ \vdots & \vdots & \vdots & 0 & \delta_{p-1} & 0 \\ 0 & 0 & 0 & 0 & 0 & 0 \end{pmatrix} .$$

The mean value in the subcompartment TR2 is given by:

$$\boldsymbol{\mu}_{TR2}(n) = E[\mathbf{Z}_{TR2}(n)] = \boldsymbol{\mu}_{TR1}(n-1)\boldsymbol{M}_{tr} \ . \qquad (12)$$

while the covariance matrix $\mathbf{S}_{TR2}(n) \in \mathbb{R}^{(p+1,p+1)}$ at time point $n$ is:

$$\mathbf{S}_{TR2}(n) = \mathbf{M}_{tr}^T \mathbf{S}_{TR1}(n-1)\mathbf{M}_{tr} + \sum_{l=1}^{p} \mathbf{V}_{tr,l}\mu_{TR1,l}(n-1) \ , \qquad (13)$$

where $\mathbf{V}_{tr,l}$ is the usual one step covariance matrix due to one cell of generation $l$ at time $n-1$.

Concerning the splitting of the cell population among the distal lymph nodes and spleen, we model this phenomenon as contemporary to the transition to the third transfer subcompartment. Hence, at the end of the third transfer time step, we are able to compute the three distinct subpopulations in the subcompartment TR3.

This step is described in the next subsection together with the proliferation in the distal nodes.

## Distal lymph nodes and spleen

The cell population evolution in the distal lymph nodes and spleen depends on the intrinsic proliferation process and the immigration flow from the transfer compartment. Since we assume that splitting of the migrating population takes place during the transition from TR2 to TR3, we can compute the three flows of cells directed to the distal compartments, directly at the level of the third transfer subcompartment. The flow from the TR2 compartment towards the distal nodes is divided into three distinct components named $\mathbf{Z}_{TR3,spl}(n), \mathbf{Z}_{TR3,il}(n), \mathbf{Z}_{TR3,mes}(n)$ to identify the number of cells addressed to the spleen, iliac and mesenteric lymph nodes, respectively. We will denote by $\rho_{spl}, \rho_{mes}, \rho_{il},\ (\rho_{il} + \rho_{mes} + \rho_{spl} = 1)$ the probabilities that a cell decides to move to the spleen, the mesenteric or the iliac node. At the same time, our model takes care of a proliferation transition during the 'splitting' time step, according to the MGW pgf modified to account for the splitting. In the remaining part of this section, we will make reference to the spleen, giving for granted that similar formulas hold for the other distal compartments. The transition function from $\mathbf{Z}_{TR2}(n)$ to $\mathbf{Z}_{TR3,spl}(n+1)$ is obtained by considering the transition matrix:

$$\mathbf{M}_{split,spl} = \rho_{spl}\mathbf{M}_{tr}\ . \tag{14}$$

Hence, we obtain:

$$\boldsymbol{\mu}_{TR3,spl}(n) = \boldsymbol{\mu}_{TR2}(n-1)\mathbf{M}_{split,spl}\ . \tag{15}$$

$$\mathbf{S}_{TR3,spl}(n) = \mathbf{M}^{T}_{split,spl}\mathbf{S}_{TR2}(n-1)\mathbf{M}_{split,spl} + \sum_{l=1}^{p}\mathbf{V}_{(split,spl),l}\mu_{TR2,l}(n-1)\ , \tag{16}$$

where $\mathbf{V}_{(split,spl),l}$ the one step covariance matrix for one cell of type $l$ at time $n-1$.

Now, we turn to the computation of the mean and the covariance matrix of cell counts in the spleen. Before doing this, we have to quantify the immigrating flow $\boldsymbol{\lambda}_{spl}(n)$ in the spleen and its covariance matrix:

$$\begin{aligned} \boldsymbol{\lambda}_{spl}(n) = E[\mathbf{Z}_{spl}(n)|\mathbf{Z}_{spl}(n-1)=0] &= \boldsymbol{\mu}_{TR3,spl}(n) \\ \mathbf{W}_{spl}(n) = Cov[\mathbf{Z}_{spl}(n)|\mathbf{Z}_{spl}(n-1)=0] &= \mathbf{S}_{TR3,spl}(n)\ . \end{aligned} \tag{17}$$

Now, we can compute the expected value of cell counts in the spleen, by considering the MGW process with an immigration flow (*43*):

$$\boldsymbol{\mu}_{spl}(n) = \boldsymbol{\mu}_{spl}(n-1)\mathbf{M}_{dist} + \boldsymbol{\lambda}_{spl}(n)\ , \tag{18}$$

where $\mathbf{M}_{dist} = \mathbf{M}_{tr}$. Finally, we can compute the covariance matrix $\mathbf{S}_{spl}(n)$ through the usual machinery, by suitably taking into account the immigrating population adding to the standard MGW process:

$$\mathbf{S}_{spl}(n) = \mathbf{M}_{dist}^T \mathbf{S}_{spl}(n-1)\mathbf{M}_{dist} + \sum_{l=1}^{p} \mathbf{V}_{tr,l}\mu_{spl,l}(n-1) + \mathbf{W}_{spl}(n) \ , \tag{19}$$

where $\mathbf{W}_{spl}(n)$ is given by (17).

# B. Model Identification

All *in vivo* CFSE experiments require to sacrifice animals to collect data. This means that measurements taken at different time points actually refer to different individuals. This fact introduces an inter-individual stochastic variability in terms of T cell counts which need be addressed in our inference scheme.

To take into account this issue, model identification has been performed by using *relative frequencies* as model variables instead of T cell counts. This choice, based on the results in ( *33, 36* ), allows one to derive an explicit expression for the asymptotic log-likelihood function of the relative frequencies when dealing only with one draining lymph node(*33, 36*). When dealing with the entire network, the inference problem becomes much more complicated. Also in our context, we take a relative frequency approach, which will allow us to derive a closed form expression for a normal approximation to the log-likelihood function. To this purpose, we introduce the vectors $\mathbf{Z}(n) = [\mathbf{Z}_{dr}(n), \mathbf{Z}^*_{spl}(n), \mathbf{Z}^*_{il}(n), \mathbf{Z}^*_{mes}(n)] \in \mathbb{R}^{p_{tot}}$ and $\boldsymbol{\mu}(n) = [\boldsymbol{\mu}_{dr}, \boldsymbol{\mu}^*_{spl}(n), \boldsymbol{\mu}^*_{il}(n), \boldsymbol{\mu}^*_{mes}(n)] \in \mathbb{R}^{p_{tot}}$ where $p_{tot} = 4p+1$ and $\mathbf{Z}^*_{spl}(n), \mathbf{Z}^*_{il}(n), \mathbf{Z}^*_{mes}(n), \boldsymbol{\mu}^*_{spl}(n), \boldsymbol{\mu}^*_{il}(n), \boldsymbol{\mu}^*_{mes}(n) \in \mathbb{R}^p$ represent the cell counts and the mean values vectors computed in the previous section, with the exclusion of the first component representing *naive* (type 0) T cells. Similarly, we introduce the covariance matrices $\mathbf{S}^*_{spl}(n), \mathbf{S}^*_{il}(n), \mathbf{S}^*_{mes}(n)$ and build up the overall cell counts covariance matrix as

$$\mathbf{S}(n) = \begin{pmatrix} \mathbf{S}_{dr}(n) & 0 & 0 & 0 \\ 0 & \mathbf{S}^*_{spl}(n) & 0 & 0 \\ 0 & 0 & \mathbf{S}^*_{il}(n) & 0 \\ 0 & 0 & 0 & \mathbf{S}^*_{mes}(n) \end{pmatrix} \in \mathbb{R}^{(p_{tot}, p_{tot})}. \tag{20}$$

We highlight that the matrix $\mathbf{S}(n)$ is introduced to incorporate the measurements taken at the different lymph nodes and spleen in the likelihood function. The block diagonal form is justified by the fact that the measurements performed in the different model compartments are independent. In order to construct the approximate likelihood function, we introduce the cell relative frequencies as $\Delta_i(n) = Z_i(n)/\sum_{i=1}^{p_{tot}} Z_i(n), i = 1, \ldots, \ p_{tot}$. Since $\sum_{i=1}^{p_{tot}} \Delta_i(n) = 1$, we consider the $p_{tot} - 1$ dimensional vector random variable $\boldsymbol{\Delta}(n) = [\Delta_1(n), \ldots, \Delta_{(p_{tot}-1)}(n)]^T \in \mathbb{R}^{p_{tot}-1}$ and the corresponding vector mean $\mathbf{r}(n) \triangleq E[\boldsymbol{\Delta}(n)]$. Moreover, we need to

compute the cell relative frequencies covariance matrix $\mathbf{\Sigma}(n) \in \mathbb{R}^{p_{tot}-1, p_{tot}-1}$. This can be carried out through manipulations similar to those in ( *29* ) (see ( *41* ) for details).

Now, assume that $c_n$ mice are sacrificed at the time point $n$. Denote by $\boldsymbol{\zeta}_k(n)$, $k = 1, 2, \ldots, c_n$, the relative frequency measurements at the timepoint $n$, and by $\mathbf{\Sigma}_k(n)$ the relative frequencies covariance matrix at the same time point. Then, the contribution to the normal approximation of the log likelihood function at time $n$ is given by:

$$L(\boldsymbol{\theta}; n) = \frac{-c_n}{2}\ln(2\pi) - \frac{1}{2}\sum_{k=1}^{c_n} log(det(\mathbf{\Sigma}_k(n)) - \frac{1}{2}\sum_{k=1}^{c_n}(\boldsymbol{\zeta}_k(n) - \mathbf{r}(n))^T(\mathbf{\Sigma}_k(n)^{-1}(\boldsymbol{\zeta}_k(n) - \mathbf{r}(n)) \ , \tag{21}$$

where $\mathbf{r}(n)$ and $\mathbf{\Sigma}_k(n)$ are functions of the probability parameter vector

$$\boldsymbol{\theta} = [\delta_0, \gamma_0, \delta_1, \gamma_1, \ldots, \delta_{p-1}, \gamma_{p-1}, \delta_p, m_1, \ldots, m_{p-1}, \rho_{spl}, \rho_{il}, \rho_{mes}]^T.$$

Of course, if independent measurements are taken at different time points $n = n_1, \ldots, n_z$, then the global negative approximate log likelihood function is

$$\mathcal{L}(\boldsymbol{\theta}) = -\sum_{i=1}^{z} L(\boldsymbol{\theta}; n_i) \ . \tag{22}$$

Notice that actually the cost function defined in (21) would represent the true log likelihood function if the relative frequencies were normally distributed: this is the case (asymptotically) when dealing with the draining lymph node only (see (*33, 36*)). In our case, the cost function (22) represents a normal approximation of the negative log likelihood function, because we have no guarantee on the asymptotic normality of relative frequencies. By minimizing the cost function function (22), we get parameter estimates:

$$\hat{\boldsymbol{\theta}} = \arg\{\min(\mathcal{L}(\boldsymbol{\theta})\} \tag{23}$$

$$\text{s.t.}$$

$$0 \le \delta_i \le 1, \ 0 \le \gamma_i \le 1, \ \delta_i + \gamma_i \le 1, \ i = 0, 1, \ldots, p-1$$

$$0 \le m_i \le 1, \ i = 1, \ldots, p-1$$

$$0 \le \delta_p \le 1,$$

$$0 \le \rho_{spl} \le 1, \ 0 \le \rho_{il} \le 1, \ 0 \le \rho_{mes} \le 1,$$

$$\rho_{spl} + \rho_{il} + \rho_{mes} = 1 \ .$$

## References

41. A. Boianelli, A. Vicino, A branching network model for T cell dissemination in adaptive immune response, Preprint. Available: arXiv:1506.02274 (2015).

42. T. E. Harris, The theory of branching processes, Courier Corporation (2002).

43. M. Quine, The Multi-Type Galton-Watson Process with Immigration, Journal of Applied Probability, **pp. 411–422** (1970).
